# Supplementary material for: Integrated decision-control for social robot autonomous navigation considering nonlinear dynamics model
Source: PLoS One. 2025 Jun 6;20(6):e0324341. doi: 10.1371/journal.pone.0324341 (PMC12143553; doi:10.1371/journal.pone.0324341)
Supplement: Attachment — Submitted filename: Response to Reviewers.docx [file pone.0324341.s002.docx]

**Integrated Decision-Control for Social Robot Autonomous Navigation Considering Nonlinear Dynamics Model**

Tracking number: PONE-D-25-07505

Dear editors and reviewers,

**On behalf of all authors, I appreciate your time and scrupulous review. It is of great importance to improve the quality of the manuscript. We highly acknowledge the contributions from the valuable reviewers. Please find the reply to your comments and suggestions point to point. Normally they are in red after your comments and suggestions.**

Yours sincerely,

Mingyue Luo

E-mail: 1202101007@stu.ccut.edu.cn

**Response to the comments by Reviewer 1**

Reviewer #1:

1: The contributions of the article are not completely clear. A more precise description of the contributions should be included.

R: Thanks for your comments. A more explicit elucidation of the contributions will facilitate readers' comprehension of the innovative aspects of the research, thereby significantly enhancing the quality of the academic paper. We have revised the introduction section by employing an itemized enumeration to further clarify the contributions of this paper. The revised parts of the paper have been pasted below for your review.

“To ensure the feasibility of the decision-control strategy for RL-based socially robots, an Integrated Decision-Control for Social Robot Autonomous Navigation (IDC-SRAN) framework that considers nonlinear dynamics models has been developed in this paper. The contributions of this paper are as follows:

- Pedestrian norm learning based on IRL. To address the issue that pedestrian walking norms are difficult to define through empirical reward functions, IRL is employed to systematically learn pedestrian walking norms. A goal-oriented expert demonstration framework is established, which integrates IRL with a Webots-based training environment for interactive exploration. This methodology enables the identification of both explicit and implicit rewards associated with pedestrian walking norms, thereby enhancing robotic social awareness in complex societal environments.
- Establishment of an Integrated Decision-Control Framework. By introducing a nonlinear dynamic model for the Four-Mecanum-Wheel Robot (FMWR), this study effectively resolves the dynamic mismatch issue prevalent in existing research. Furthermore, the system's actions are defined as incremental torques for the four wheels, which can be directly applied to actual robotic control units. This approach achieves an integrated decision-control for robot autonomous navigation, while enhancing the feasibility of decision-control strategies.
- Design of Driving-Force-Guided Reward Function. Considering the challenge posed by state delays resulting from transient characteristics of the model in reward function design, this study innovatively integrates features of driving force into the reward mechanism. By rewarding driving forces aligned with correct directions, the framework guides robots to conduct proper exploration during transient model phases. This approach ensures task efficiency and safety while significantly reducing the occurrence of suboptimal behaviors.”

2: The choice of the Four-Mechanism-Wheel Robot (FMWR) for the experiments is unjustified.

R: Thanks for your comments. We further explained the reasons and significance of choosing the Four Mechanics Wheel Robot in the paper, it has been instrumental in refining our paper. The revised parts of the paper have been pasted below for your review.

“This study focuses on how to integrate wheeled service robots into human social scenarios to provide services. To enhance the social awareness of service robots, it is necessary to simulate the movement patterns of pedestrians, which are characterized by omni-directionality. Therefore, selecting the FMWR, which is capable of omni-directional movement, as the research object is highly meaningful.”

3: Line 159 is unclear; something is missing after the colon.

R: Thanks for your comments. The description of this part in the previous version of this paper was indeed insufficiently clear. We have revised the relevant content, which has been pasted below for your review.

“The feasibility challenge of goal-oriented, socially-aware robot integrated decision-control can be articulated as follows: Given an initial and a target state, the robot must devise decisions that conform to pedestrian walking norms and execute actions conforming to model dynamics, thereby ensuring a smooth transition from the initial state to the target state.”

4: In equations 19 and 20, elements are not separated by the appropriate space, and the words used to express the cases are not understood.

R: Thank you for your meticulous examination. We have rearranged the formulas and further explained the meanings of these two formulas. The revised parts of the paper have been pasted below for your review.

$R_{o-x}=\left\{\begin{matrix} -|\vec{F}_{x}|, & v_{x}(x-x_{i})<0\text{ and }v_{x}\cdot F_{x}>0 \\ |\vec{F}_{x}|, & v_{x}(x-x_{i})<0\text{ and }v_{x}\cdot F_{x}<0 \end{matrix}\right.$ (19)

$R_{o-y}=\left\{\begin{matrix} -|\vec{F}_{y}|, & v_{y}(y-y_{i})<0\text{ and }v_{y}\cdot F_{y}>0 \\ |\vec{F}_{y}|, & v_{y}(y-y_{i})<0\text{ and }v_{y}\cdot F_{y}<0 \end{matrix}\right.$ (20)

“Here $R_{o-x}$ denote the reward function for the robot's lateral avoidance of obstacles. When the robot's longitudinal velocity relative to the pedestrian is in the opposite direction of the relative distance, i.e., $v_{x}(x-x_{i})<0\text{ }$, it indicates that the robot is moving towards the pedestrian. In this case, if the velocity component $v_{x}$ and the force $F_{x}$ are in the same direction, i.e., $v_{x}\cdot F_{x}>0$, it suggests that the robot's motion towards the pedestrian is accelerating, which may pose a collision risk or cause discomfort to the pedestrian. The system will then impose a penalty. Conversely, if the velocity component $v_{x}$ and the force $F_{x}$ are in opposite directions, i.e., $v_{x}\cdot F_{x}<0$, it indicates that the control system is effectively implementing deceleration to avoid the pedestrian, and the system will provide a positive reward. Similarly, a reward function $R_{o-y}$ has been designed for robots to lateral avoid obstacles.”

5: The experiments carried out in simulation do not mention the limitations of the simulated environment.

R: Thanks for your comments. In the initial version, the manuscript neglected to mention the limitations of the simulated environment. We have supplemented the relevant explanations in the revised version, with the added content provided below for your review.

“It's no doubt that this compromise validation approach has limitations: it does not account for unknown disturbances in sensor perception and overlooks discrepancies between simulated conditions (robot models, pedestrian kinematics, environments) and real-world implementations. These gaps will be addressed in future research.”

6: Advantage Actor-Critic (A2C) is used for decision-making, but it is not justified why it is chosen over other reinforcement learning strategies, such as PPO or SAC, which are also popular in autonomous navigation problems.

R: Thanks for your comments. We further explained the reasons and significance of choosing the Advantage Actor-Critic in this paper. The revised parts of the paper have been pasted below for your review.

“Considering the inherent advantages of the Advantage Actor-Critic (A2C) algorithm, which enables parallel synchronous updates and typically achieves higher training efficiency with fewer hyperparameters, and it avoids hyperparameters adjustments associated with Proximal Policy Optimization (PPO) clipping ranges or entropy coefficient balancing in Soft Actor-Critic (SAC). To optimize both computational efficiency and hyperparameters adjustments simplicity, the A2C is employed to construct the IDC-SRAN framework. The advantage function of A2C can be mathematically expressed as,”

**Response to the comments by Reviewer 2**

Reviewer #2: This article presents a valuable contribution to social robot autonomous navigation through a novel Integrated Decision-Control Framework for Social Robot Autonomous Navigation (IDC-SRAN) that considers nonlinear dynamics models. The work addresses the important challenge of ensuring reinforcement learning strategy feasibility in real-world applications by bridging the dynamics mismatch between simulations and physical implementation. Although the methodology is well-conceived and demonstrates promising results, several aspects of the presentation, evaluation, and implementation details need significant improvement before publication. My comments are as follows:

1- The abstract effectively conveys the core contribution of the paper but contains several grammatical issues and redundancies. For example, "but lacks discussions on the feasibility of strategies" could be more precisely stated. The phrase "which takes into account the nonlinearity of the social robot model" appears twice in different forms. The technical approach is well summarized, but I recommend revising for clarity and conciseness. The statement about experiments "demonstrating intelligence and feasibility" is vague - specific metrics of improvement would strengthen this claim.

R: Thanks for your comments. A clearer and more concise articulation of the abstract will enhance readers' comprehension of the research content and innovative aspects, thus significantly improving the quality of the academic paper. We have revised the grammar and expressions in the abstract section, and the amended portions of the manuscript have been appended below for your review.

“Reinforcement learning (RL) has demonstrated significant potential in social robot autonomous navigation, yet existing research lacks in-depth discussion on the feasibility of navigation strategies. Therefore, this paper proposes an Integrated Decision-Control Framework for Social Robot Autonomous Navigation (IDC-SRAN), which accounts for the nonlinearity of social robot model and ensures the feasibility of decision-control strategy. Initially, inverse reinforcement learning (IRL) is employed to tackle the challenge of designing pedestrian walking reward. Subsequently, the Four-Mecanum-Wheel Robot dynamic model is constructed to develop IDC-SRAN, resolving the issue of dynamics mismatch of RL system. The actions of IDC-SRAN are defined as additional torque, with actual torque and lateral/longitudinal velocities integrated into the state space. The feasibility of the decision-control strategy is ensured by constraining the range of actions. Furthermore, a critical challenge arises from the state delay caused by model transient characteristics, which complicates the articulation of nonlinear relationships between states and actions through IRL-based rewards. To mitigate this, a driving-force-guided reward is proposed. This reward guides the robot to explore more appropriate decision-control strategies by expected direction of driving force, thereby reducing non-optimal behaviors during transient phases. Experimental results demonstrate that IDC-SRAN achieves peak accelerations approximately 8.3% of baseline methods, significantly enhancing the feasibility of decision-control strategies. Simultaneously, the framework enables goal-oriented autonomous navigation through active torque modulation, attaining a task completion rate exceeding 90%. These outcomes further validate the intelligence and robustness of the proposed IDC-SRAN.”

2- The introduction provides adequate background on RL for robot navigation but could benefit from a more focused discussion of the specific gap being addressed.

R: Thanks for your comments. we have conducted in-depth discussions and systematically restructured the introduction section. The specific improvements are as follows.

1. We have analyzed the limitations of traditional navigation paradigms in open social scenarios. By introducing references, we have strengthened the theoretical basis for the necessity of adopting a reinforcement learning framework.

“Autonomous navigation, as a core technology for service robots, logistics robots, and other intelligent agents to integrate into human society, is undergoing a paradigm shift from structured environments to dynamic social scenarios, facing the challenge of safe navigation in densely populated environments. Traditional navigation methods based on predefined rules and deterministic optimization, while offering distinct advantages in the interpretability of path generation, often struggle to effectively handle the random behaviors of pedestrian movements [3,4]. The decision freeze represents a fundamental limitation of such approaches in dynamic environments [5,6]. To break through this bottleneck, scholars have proposed an innovative direction of integrating reinforcement learning with traditional planning [7,8]. For example, Pérez et al. [9] by using global path planning to provide waypoint guidance and combining reinforcement learning to handle local pedestrian interactions, the integration of dynamic speed adjustment mechanisms and composite reward functions has successfully addressed the issue of low navigation efficiency in constrained scenarios.”

[3] Wang B, Liu Z, Li Q, & Prorok, A. Mobile robot path planning in dynamic environments through globally guided reinforcement learning. IEEE Robotics and Automation Letters, 2020, 5(4), 6932-6939.
[4] Shi H, Yu W, Madani K. A Review on Social Awareness Navigation for Service Robots. InInternational Conference on Social Robotics, 2024, 143-152.
[5] Zhang P, Chen J J, Jin D, & Jung, S. Service robots in crowded environments: How crowd dynamics shape robotic adoption intention at events, Journal of Hospitality and Tourism Management, 2024, 61, 251-260.
[6] Turkle S. A nascent robotics culture: New complicities for companionship. In Machine ethics and robot ethics Routledge, 2020, 107-116.
[7] Francis A, Pérez-d’Arpino C, et al. Principles and guidelines for evaluating social robot navigation algorithms. ACM Transactions on Human-Robot Interaction, 2025, 14(2), 1-65.
[8] Singamaneni P T, Bachiller-Burgos P, et al. A survey on socially aware robot navigation: Taxonomy and future challenges. The International Journal of Robotics Research, 2024, 43(10), 1533-1572.
[9] Pérez-D’Arpino C, Liu C, et al. Robot navigation in constrained pedestrian environments using reinforcement learning. International Conference on Robotics and Automation (ICRA), 2021, 1140-1146.

2. Based on a comprehensive review of reinforcement learning applications in fields such as micro-robots and space robots, this paper reveals the crucial role of dynamics modeling in enhancing the effectiveness of robotic reinforcement learning decision-making and control strategies. The accuracy of the dynamics modeling directly affects the environmental adaptability and task completion robustness of autonomous intelligent systems.

“As proposed by Heuthe et al. in Science Robotics, in the research on microrobot swarm control, counterfactual reward mechanisms must be combined with intrinsic dynamics modeling to achieve precise torque control [29]. The latest research published in Acta Astronautica indicates that dynamics simulation based on the MuJoCo physics engine is crucial for ensuring the stability of space robot grasping control [30]. Several surveys on reinforcement learning in robotic applications have also confirmed that accurate reproduction of dynamic models is a necessary condition for solving the sim2real problem [31,32].”

[29] Heuthe VL, Panizon E, Gu H, Bechinger C. Counterfactual rewards promote collective transport using individually controlled swarm microrobots. Science Robotics. 2024, 18, 9(97): eado5888.
[30] Jahanshahi H, Zhu ZH. Review of machine learning in robotic grasping control in space application. Acta Astronautica. 2024, 15.
[31] Nguyen H, La H. Review of deep reinforcement learning for robot manipulation. international conference on robotic computing (IRC), 2019, 590-595.
[32] Singh B, Kumar R, Singh VP. Reinforcement learning in robotic applications: a comprehensive survey. Artificial Intelligence Review. 2022, 55(2):945-90.

3- Lines 35-49: The discussion on nonlinear characteristics and transient behaviors is relevant but somewhat disconnected from the preceding paragraphs. Consider restructuring to create a more coherent narrative.

R: Thanks for your comments. Coherent expression contributes to the logical integrity and continuity of the introduction, thereby elevating the overall quality of the manuscript. We have revised the introduction by enhancing the articulation of logical connections, and the amended sections of the manuscript are appended below for your review.

“Several surveys on reinforcement learning in robotic applications have also confirmed that accurate reproduction of dynamic models is a necessary condition for solving the sim2real problem. It is commonly believed that the accuracy of a model gradually increases with the number of degrees of freedom. Meanwhile, the nonlinear relationships between different degrees of freedom also intensify the transient characteristics among the models. During the transient transition process, due to the time lag of the state, there may be significant discrepancies between the trend of change and the actions, which can lead to erroneous reward signals.”

4- The contributions should be more explicitly stated, perhaps as bullet points, to help readers identify the novelty.

R: Thanks for your comments. A more explicit elucidation of the contributions will facilitate readers' comprehension of the innovative aspects of the research, thereby significantly enhancing the quality of the academic paper. We have revised the introduction section by employing an itemized enumeration to further clarify the contributions of this paper. The revised parts of the paper have been pasted below for your reference.

“To ensure the feasibility of the decision-control strategy for RL-based socially robots, an Integrated Decision-Control for Social Robot Autonomous Navigation (IDC-SRAN) framework that considers nonlinear dynamics models has been developed in this paper. The contributions of this paper are as follows:

- Pedestrian norm learning based on IRL. To address the issue that pedestrian walking norms are difficult to define through empirical reward functions, IRL is employed to systematically learn pedestrian walking norms. A goal-oriented expert demonstration framework is established, which integrates IRL with a Webots-based training environment for interactive exploration. This methodology enables the identification of both explicit and implicit rewards associated with pedestrian walking norms, thereby enhancing robotic social awareness in complex societal environments.
- Establishment of an Integrated Decision-Control Framework. By introducing a nonlinear dynamic model for the Four-Mecanum-Wheel Robot (FMWR), this study effectively resolves the dynamic mismatch issue prevalent in existing research. Furthermore, the system's actions are defined as incremental torques for the four wheels, which can be directly applied to actual robotic control units. This approach achieves an integrated decision-control for robot autonomous navigation, while enhancing the feasibility of decision-control strategies.
- Design of Driving-Force-Guided Reward Function. Considering the challenge posed by state delays resulting from transient characteristics of the model in reward function design, this study innovatively integrates features of driving force into the reward mechanism. By rewarding driving forces aligned with correct directions, the framework guides robots to conduct proper exploration during transient model phases. This approach ensures task efficiency and safety while significantly reducing the occurrence of suboptimal behaviors.”

5- The literature review misses some key recent works on dynamics-aware RL for robotics and sim-to-real transfer, particularly those published in 2023-2025.

R: Thanks for your comments. We have revised the Introduction section to incorporate recent advancements in dynamics-aware RL for robotics and sim-to-real transfer, including several pivotal studies published in 2023-2025. The revised parts of the paper have been pasted below for your reference.

“In addition, recent studies have enhanced the feasibility of sim2real transfer through perceptual methods [34,35]. For instance, the bird's-eye view semantic conversion method proposed by Reiher [36], and the RetinaGAN image adaptation technique proposed by Ho [37] have demonstrated significant effectiveness.”

[34] Li J, Yang Z, Wang X, et al. Jotr: 3d joint contrastive learning with transformers for occluded human mesh recovery. In Proceedings of the IEEE/CVF International Conference on Computer Vision, 2023, 9110-9121.
[35] Shin J, Jeong H, Rameau F, Kum D. Instagram: Instance-level graph modeling for vectorized hd map learning. IEEE Transactions on Intelligent Transportation Systems, 2025.
[36] Reiher L, Lampe B, Eckstein L. A sim2real deep learning approach for the transformation of images from multiple vehicle-mounted cameras to a semantically segmented image in bird’s eye view. International Conference on Intelligent Transportation Systems (ITSC), 2020, 1-7.
[37] Ho D, Rao K, Xu Z, et al. Retinagan: An object-aware approach to sim2real transfer. International Conference on Robotics and Automation (ICRA), 2021, 10920-10926.

6- The introduction provides a good overview of reinforcement learning for robot navigation, but could benefit from incorporating insights from related domains where similar challenges have been addressed. The recent reviews like "Reinforcement learning in robotic applications: a comprehensive survey", "Review of deep reinforcement learning for robot manipulation", "Review of machine learning in robotic grasping control in space application" discusses challenges in bridging simulation-to-reality gaps in robotic control systems, which parallels your focus on dynamics mismatch issues. This reference would strengthen your discussion in lines 37-39 about the "significant gap from simulation to reality (sim2real)" and provide readers with additional context on how similar challenges manifest across different robotic applications.

R: Thank you for your comments. We have revised the introduction section by incorporating methodological insights from reinforcement learning applications in space robotics, microrobotics, and related domains. Additionally, we have supplemented new references and significantly strengthened the discussion on "the impact of dynamics models on sim2real performance." The updated manuscript sections are appended below for your review.

“The core of current research in social robots lies in learning the walking norms of pedestrians to enhance the robots' adaptability and flexibility in social environments. However, the RL methods often simplify the problem, neglecting the dynamics mismatch between pedestrians and robots. These methods overlook the mechanical properties and employ simplistic kinematic models to describe the motion states of robots, which results in a significant sim2real gap [28]. As proposed by Heuthe et al. in Science Robotics, in the research on microrobot swarm control, counterfactual reward mechanisms must be combined with intrinsic dynamics modeling to achieve precise torque control [29]. The latest research published in Acta Astronautica indicates that dynamics simulation based on the MuJoCo physics engine is crucial for ensuring the stability of space robot grasping control [30]. Several surveys on reinforcement learning in robotic applications have also confirmed that accurate reproduction of dynamic models is a necessary condition for solving the sim-to-real problem [31,32]. However, the incorporation of such models introduces significant challenges in RL system design: including the formulation of empirical reward functions accounting for historical states, and issues pertaining to system convergence [33].”

[28] Kong W, Zhou D, Yang Z, Zhao Y, Zhang K. UAV autonomous aerial combat maneuver strategy generation with observation error based on state-adversarial deep deterministic policy gradient and inverse reinforcement learning. Electronics. 2020, 9(7):1121.
[29] Heuthe VL, Panizon E, Gu H, Bechinger C. Counterfactual rewards promote collective transport using individually controlled swarm microrobots. Science Robotics. 2024, 18, 9(97): eado5888.
[30] Jahanshahi H, Zhu ZH. Review of machine learning in robotic grasping control in space application. Acta Astronautica. 2024, 15.
[31] Nguyen H, La H. Review of deep reinforcement learning for robot manipulation. In2019 Third IEEE international conference on robotic computing (IRC), 2019, 590-595.
[32] Singh B, Kumar R, Singh VP. Reinforcement learning in robotic applications: a comprehensive survey. Artificial Intelligence Review. 2022, 55(2):945-90.
[33] Nayeem R, Bazzi S, et al. Transient behavior and predictability in manipulating complex objects. International Conference on Robotics and Automation (ICRA), 2020, 10155-10161.

7- The paper's hierarchical structure is unclear in several places, making it difficult to follow the logical flow of ideas. I recommend implementing a consistent numbering system (e.g., 1, 1.1, 1.1.1) or clear typographical distinctions between section levels to improve readability and help readers navigate the paper's structure.

R：Thank you for your suggestion. We acknowledge that the initial version overlooked this critical issue. The revised version now implements comprehensive modifications that significantly enhance the organizational structure and logical coherence of the manuscript.

8- The kinematic model in Equation (1) is a reasonable foundation, but the analysis would benefit from explaining the practical implications of these equations for real-world control. How do errors in this model affect controller performance?

R: We sincerely appreciate your valuable suggestions regarding the practical-theoretical alignment of the kinematic model. In response to the critical methodological concerns you raised, we have systematically incorporated supplemental analyses addressing these points in Section 2.1 of the revised manuscript. The updated manuscript sections are appended below for your review.

“Considering the kinematic model error adversely affect the precision of state transitions in reinforcement learning (RL) systems, thereby affecting controller performance. Consequently, establishing high-fidelity dynamic models constitutes a critical step to mitigating the sim2real gap.”

9- The mass matrix M(q) in Equation (5) assumes uniform mass distribution, but real robots often have asymmetric mass distributions. This simplification should be acknowledged as a potential limitation.

R：Thank you for your comment. The assumption of uniform mass distribution is a potential limitation within our constructed model. In response, we have explicitly acknowledged this limitation in the revised manuscript and specifically highlighted in the conclusions section as a critical direction for future research to enhance model precision through more comprehensive and detailed discussions of dynamic characteristics. The revised content is provided below for your evaluation.

“To streamline the model formulation, this paper postulates a uniform mass distribution for the robot, which is a potential limitation that will be the focus in the future research.”

10- The Coriolis and centrifugal force matrix C(q,q̇) in Equation (6) includes terms for platform rotation but doesn't address how wheel-surface interactions might affect these forces during high-speed maneuvers.

R: Thank you for your comment. Considering that the core innovation of this study lies in demonstrating the feasibility enhancement of decision-control strategies through model integration, rather than the complexity of the model itself, certain simplifications were introduced during the modeling process. This methodological trade-off constitutes a potential limitation of our proposed framework. To maintain full transparency, the relevant declarations regarding these simplifications have been incorporated into the revised manuscript as follows.

“This paper ignores the influence of the wheel-surface interactions on Coriolis and centrifugal forces, which are expressed as:”

11- Equation (8) introduces the nominal mass term M0, but doesn't discuss how the uncertainty in mass estimation affects control stability, which is critical for reliable navigation.

R：Thank you for your comment. The explanatory content regarding this aspect has been supplemented in the revised manuscript. The revised content is provided below for your evaluation.

“This paper focuses on pedestrian movement imitation of robot, where motion control systems demonstrate strong stability in low-velocity regimes (consistent with typical pedestrian locomotion speeds). Consequently, the unknown component of mass is considered to be of negligible magnitude.”

12- The state space representation (lines 164-177) includes robot and pedestrian positions/velocities but doesn't explicitly include uncertainty measurements, which are crucial for robust decision-making in dynamic environments.

R: We sincerely appreciate your valuable feedback on our manuscript. Your insightful recommendation regarding the explicit consideration of uncertainty quantification in state-space representation holds significant implications for enhancing the robustness of decision-making systems in dynamic environments. In response to your suggestion, we have incorporated explicit clarifications in Section 3.1 of the revised manuscript as follows:

“This paper assumes all states to be fully observable without unknown disturbances at the perceptual level. This idealized assumption is formulated to facilitate clearer investigation of the model's impact on decision-control feasibility, with refinements to be addressed in subsequent research efforts.”

We reiterate our gratitude for your expert comments, which have enabled us to more precisely delineate the boundary conditions and applicability scope of this research. The uncertainty modeling challenges you identified will constitute a central focus of our subsequent research initiatives.

13- The reward function learning approach using IRL is interesting, but the explanation of how the GOED dataset was created lacks sufficient detail - particularly about how final pedestrian positions were determined to be "goal positions" when this information wasn't explicitly available in the ETH dataset.

R: We sincerely appreciate your insightful comments regarding the dataset construction methodology. In response to the critical issue of "goal positions" inference you highlighted, we have provided a comprehensive elaboration in Section 3.2 of the revised manuscript, supplemented with illustrative diagrams to enhance conceptual clarity. The updated sections of the manuscript and the additional figure are appended below for your review.

“In the execution of tasks, robots require a clear goal point. However, in real-world scenarios, the destinations of pedestrians are often unknown, which implies that existing expert demonstration data lacks information pertaining to goal-directed behaviour. Therefore, the states of GOED, in addition to their own information, should also include all details of the goal, pedestrians, and static obstacles. This paper employs the ZARA scenarios from the ETH dataset as expert demonstration samples, which provide pedestrian coordinate information at intervals of 0.4 s. In the experimental setup, a pedestrian from the scene is randomly selected as the target agent. Figure 4 shows a frame of its motion process. The current position of the agent is marked with a red circle, while its trajectory and terminal coordinates are represented by the red dashed line and solid dot, respectively. The boundary box outlined by the light blue dashed lines quantifies the spatial distance between the agent and the scene's edges. Based on the nearest distance principle, the four pedestrians most closely interacting with the agent are selected and their current positions are marked with green, yellow, pink, and orange circles, respectively. The trajectories and terminal coordinates of these interacting objects are represented by the dashed lines and solid dots in the corresponding colors. It is worth noting that the pedestrian targets are unknown in real-world scenarios. However, in the limited environment of the TE, defining the terminal coordinates within the scene as the task goal is engineeringly reasonable. This approach is consistent with the logic of autonomous driving systems, which preset waypoints in known topological map [40]. In this manner, an expert demonstration dataset containing target point information, referred to as the GOED, is constructed. This approach is pivotal for the IRL process, which is employed to learn the underlying reward functions that reflect pedestrian walking norms and to enhance the performance of robots in complex social environments.”

Figure 4 The ZARA scenarios from the ETH for GOED

[40] Huang H, Liu Y, Liu J, Yang Q, Wang J, Abbink D, Zgonnikov A. General optimal trajectory planning: Enabling autonomous vehicles with the principle of least action. Engineering}, 2024, 33, 63-76.

14- The CIIRL framework mentioned on line 184 is not clearly defined before being used.

R: We sincerely appreciate your feedback and regret the omission of the CIIRL framework definition in the initial manuscript. We have revised Section 3.2 of the article to incorporate a clear definition of CIIRL. Thank you again for your meticulous review, which has been invaluable in improving our paper. The updated manuscript sections are appended below for your review.

“This section describes the establishment of the Collaborative Interactive Reverse Reinforcement Learning (CIIRL) framework, which utilizes Maximum Entropy Deep Inverse Reinforcement Learning (MEDIRL) combined with GOED and Training Environment (TE) to explore pedestrian motion norms, i.e., to explore the social reward function of pedestrian interactive motion. The goal of robots is to complete tasks in a safe and effective manner. Imitating human behavior patterns helps to improve the acceptability of their behavior.”

15- The relationship between the Maximum Entropy Deep Inverse Reinforcement Learning (MEDIRL) in line 223 and the previously mentioned IRL approach isn't clearly established.

R: We sincerely appreciate your feedback and regret any misunderstandings that may have arisen from insufficient clarity in the initial manuscript description. In this study, the inverse reinforcement learning (IRL) methodology employed is specifically the Maximum Entropy Deep Inverse Reinforcement Learning (MEDIRL). We have revised Section 3.2 of the article to clarify that the adopted IRL approach corresponds precisely to the MEDIRL methodology. The updated manuscript sections are appended below for your review.

“This section describes the establishment of the Collaborative Interactive Reverse Reinforcement Learning (CIIRL) framework, which utilizes Maximum Entropy Deep Inverse Reinforcement Learning (MEDIRL) combined with GOED and Training Environment (TE) to explore pedestrian motion norms, i.e., to explore the social reward function of pedestrian interactive motion. The goal of robots is to complete tasks in a safe and effective manner. Imitating human behavior patterns helps to improve the acceptability of their behavior.”

16- The driving-force-guided reward function in Equation (18) is a novel contribution, but there's no ablation study showing its specific impact compared to alternative formulations.

R：Thank you for your comments. We sincerely apologize for the misunderstanding caused by insufficient clarity in the initial manuscript version. In fact, the driving-force-guided reward function encompasses two components: one corresponding to the goal and another to obstacles (including both static obstacles and dynamic pedestrians). The ablation study comparing with and without driving-force-guided reward function is presented in Section 3.5 (Figure 8). Thank you again for your meticulous review, as it has been instrumental in refining our paper. We have carefully revised the sections with ambiguous phrasing, and the revised content is provided below for your evaluation.

“In summary, the reward function R of IDC-SRAN can expressed as,

$R=R_{f}+c_{3}R_{coll}+c_{4}R_{com}+c_{5}R_{s}$ (24)

$R_{f}=c_{1}R_{G}+c_{2}R_{o}$ (25)

Where $C_{i}$ are referred to as the reward weighting constants.

In Figures 8(a) and Figures 8(b), the blue lines distinctly illustrate the training loss under the IDC-SRAN framework, encompassing both policy loss and value loss as pivotal metrics. In parallel, the orange lines depict the training loss of the baseline IDC framework without deploying R_f_, maintaining identical experimental settings.”

17- The constraint of actions to [-1,1] range (line 181) seems arbitrary - the physical justification for this specific range should be explained.

R: We appreciate your feedback. We apologize any misunderstandings that may have arisen from insufficient clarity in the initial manuscript description. In practice, analyses accounting for the system's discrete time-step parameters and related factors confirm that the [-1,1] range constitutes an acceptable operational specification for the majority of robotic control units. We have expanded the relevant content in Section 3.1 of the manuscript, with the updated materials provided below for your reference.

“By restricting the range of the additional torques to [-1,1], the feasibility of the decision-control strategy is ensured. Given the system's discrete step size of 0.02 seconds, the action range of ±1 limits the torque rate variation to below 50 Nm/s, a specification that is acceptable for the vast majority of robotic control units.”

18- The paper lacks details on the simulator parameters - particularly how the Webots environment was configured to represent realistic pedestrian behaviors.

R：Thank you for your comments. The initial manuscript version indeed omitted the technical details on the simulator parameters. In the revised version, we have incorporated a new section that provides comprehensive elaboration of environmental details, facilitating readers' comprehension of pedestrian behavioral patterns within the simulated environment as well as its specific environmental parameters. The revised parts of this paper have been pasted below for your review.

“To train the proposed IDC-SRAN framework and validate its autonomous navigation capabilities, this study leveraged the Webots simulation platform to construct a testing and validation environment that replicates the real-world environment of the ETH dataset, as illustrated in Figure 6.”

Fig 6. Simulation Environment for Testing and Validation (Scene 1). (a) Real-World Environment of the ETH Dataset. (b) Simulation Environment with Webots

Figure 6(a) depicts the real-world environment of the ETH dataset, and Figure 6 (b) presents the testing and validation simulation environment with the Webots simulation platform. The white lines represent walls enclosing a rectangular structure in the lower-left area, resembling commercial facilities in real-world environments with pedestrian-impermeable boundaries. It is noteworthy that the simulation environment does not constitute a 1:1 replication of the real-world environment. Specifically, parked vehicles along road edges are treated as pedestrian-impermeable boundaries in the simulation, and the depth of the lower-right corner is deliberately deepened. This design modification reduces corner width while increasing its length, thereby enhancing the operational complexity for robots to pass through the corner. Such modification further facilitate the training and validation of rationality in autonomous navigation decision-control strategies. The simulated environment measures 21.6 meters in width and 17.4 meters in length, with the commercial facilities in the lower-left area measures 15.5 meters in width and 7.7 meters in length, containing a total of 15 pedestrians.

Fig 7. Additional verification environment for Real-World Corridor (Scene 2)

Furthermore, this study is grounded in the assumption of global observability, neglecting the research on the perception module. Consequently, IDC-SRAN remains constrained to simulation environments and faces significant challenges in practical deployment on real-world robotic platforms. Notably, limited scene generalization capability emerges as one of the critical obstacles in practical implementation of autonomous navigation decision-control systems. Therefore, an additional verification environment was developed based on existing conditions, which is shown in Figure 7, precisely aligned with a real-world corridor to validate the generalization performance of IDC-SRAN. The additional verification environment features a rectangular configuration with a length of 12 meters and a width of 8.5 meters, containing a total of 10 pedestrians. The movement of all pedestrians within the environment is governed by the social force model, which continuously calculates their speed and position at each discrete time step. Motion control in the simulated environment is ultimately executed through the Supervisor node of the Webots platform, enabling precise simulation of pedestrian dynamics. ”

19- The comparative analysis between ADM-RL and IDC-SRAN frameworks is qualitative rather than quantitative - no statistical measures of performance improvement are provided.

R：Thank you for your comments. We sincerely apologize that in the initial manuscript version, comprehensive and meticulous consideration was not given during the experimental phase. The absence of quantitative metrics resulted in overly ambiguous analytical outcomes, which compromised the quality of our work. In the revised manuscript, we have added bar charts illustrating the mean and maximum acceleration values (Figure 13), along with comparing collision rates and completion rates across different scene (Table 1). These statistical additions will provide readers with clearer insights into the core contributions of the study. Once again, we sincerely appreciate your valuable feedback, which has been instrumental in enhancing the manuscript's quality. Due to the wide coverage of the revised content, please refer to Chapter 4 of the manuscript for specific details.

20- The policy and value network architecture description (lines 295-298) is too brief - more details on layer sizes, activation functions, and training hyperparameters are needed for reproducibility.

R：Thank you for your comments. Additional parameter details regarding the policy and value networks have been supplemented in the revised manuscript. The revised parts of this paper have been pasted below for your review.

“The policy and value networks consist of a three-layer DNN, each with 128 neurons in the hidden layers. Among them, both the policy network and value network feature an input layer dimension of 30, representing the number of states in the IDC-SRAN framework. Their output layer dimensions of 4 and 1 correspond to the action number and the policy evaluation value, respectively. ReLU activation functions are used across all networks, and the optimizer is Adam with a weight decay of 10^-4^. The learning rates are 5×10^-5^ for the reward network and 10^-4^ for A2C, the discount rate is 0.99.”

21- The training process description doesn't specify the number of training iterations, computational resources used, or convergence criteria.

R：Thank you for your comments. Additional details regarding the training process have been incorporated into the revised manuscript. The revised parts of this paper have been pasted below for your review.

“During training, iterative updates were performed every 3000 steps, with a convergence criterion defined as the average of policy and value losses over ten consecutive iterations being less than 1.”

22- Figures 5(a) and 5(b) show training losses, but don't include validation losses, making it difficult to assess potential overfitting.

R：Thank you for your comments. The inclusion of training losses-related curves enables the evaluation of potential overfitting in the IDC-SRAN framework while providing clearer insights into its convergence behavior and stability. Therefore, the revised manuscript presents training losses-related curves derived from the total average return during the evaluation phase after every iteration. The revised parts have been pasted below for your review.

“Furthermore, to further validate training efficacy, the Total Average Return (TAR) is presented in Figure 9, which is derived from the evaluation phase after every iteration. While the TAR trajectory of the IDC framework without R_f_ exhibits a gradually increasing trend, it is accompanied by pronounced oscillations. Combined with the loss oscillations illustrated in Figure 8 demonstrate that state delay caused by transient characteristics of robot model hinders the agent's to correctly associate rewards with the actions generating them. By contrast, the introduction of R_f_ enables rational exploration during model transients, leading to marked improvements in both stability and convergence.”

23- The velocity profiles in Figures 6(b) and 7(b) show qualitative improvements in smoothness, but lack quantitative metrics such as jerk measurements or energy consumption comparisons.

R：Thank you for your comments. In the initial manuscript version, we indeed overlooked the inclusion of quantitative metrics demonstrating improvements in velocity smoothness. To address this, the revised manuscript specifically incorporates acceleration curves alongside bar charts illustrating peak and mean acceleration values. These additions enable readers to gain intuitive insights into the enhanced feasibility of decision-control strategies by the IDC-SRAN framework through quantifiable visual representations. The revised parts have been pasted below for your review.

Fig 12. The accelerations of ADM-RL and IDC-SRAN in Scene 1.

To quantitatively analyze the impact of IDC-SRAN and ADM-RL on velocity smoothness, acceleration-related data from different algorithms in Scenario 1 are presented in Figures 12 and 13. The acceleration profile of ADM-RL illustrated in Figures 12(a), after entering the crowded area, the robot exhibited drastic velocity fluctuations while maneuvering to avoid pedestrians and proceed toward the goal. Notably, most velocity adjustments were executed within a single discrete time step, resulting in frequent sudden acceleration changes. While these actions demonstrate the intelligence of decision-control, effectively preventing collisions and maintaining correct goal-oriented movement, their practical implementation faces limitations due to actuator dynamic response constraints, thereby compromising the feasibility of realizing such actions in real-world scenarios. Figures 13 the bar chart comparing the mean and maximum absolute acceleration of both IDC-SRAN and ADM-RL. Under identical scenarios, IDC-SRAN demonstrates notably rational acceleration characteristics, with its maximum instantaneous acceleration capped at 1, which is only 8.3% of the peak value by ADM-RL. This substantial reduction in acceleration extremes not only validates the feasibility of IDC-SRAN's decision-control strategy but also enhances pedestrian interaction experience, as smooth acceleration transitions help prevent startling pedestrians. This outcome further corroborates the intelligence of IDC-SRAN in adaptive control and autonomous navigation of robot.

Fig 13. The Bar Chart Comparing The Mean and Maximum Absolute Acceleration of Both IDC-SRAN and ADM-RL

24- The robot trajectories in Figures 6(a) and 7(a) demonstrate different behaviors, but there's no analysis of path optimality or safety margins.

R：Thank you for your comments. The revised manuscript provides further analysis of the optimality in robot path, and the revised parts have been pasted below for your review.

However, as a decision-control scheme, it eliminates the need for real-time prediction of dynamic pedestrians' behaviors or optimal path planning. This design results in a compromise in path optimality compared to conventional planning-based tracking algorithms. Nonetheless, this does not imply that the path optimality of the decision-making control approach cannot be improved, which will be further studied in future research.

25- The robot-pedestrian distance measurements in Figure 9(a) show the robot maintains distances above the "intimate distance" threshold, but there's no statistical analysis of how consistently these safety distances are maintained.

R：Thank you for your comments. In the revised manuscript, we have further elaborated on this section to ensure comprehensive coverage of details. The revised parts have been pasted below for your review.

Compared to robots that remain stationary, those that adjust their velocity to avoid dynamic pedestrians exhibit behaviors more akin to natural human evasive habits. Figure 15 illustrates the relative distance variations between the robot and the four nearest pedestrians, with blue and red dashed lines demarcating the boundaries of comfortable and intimate distances as per human social norms, which are 1.2 m and 0.5 m, respectively. Due to the fact that the distance sequence of pedestrians relative to robots in the simulated environment constantly changes between two adjacent moments, Figure 15 presents a discrete point form. During the approximately 10–12-second interval, the nearest pedestrian progressively approached the robot. To avoid excessively compromising the pedestrian’s comfort boundaries, the robot proactively reduced its lateral velocity while increasing longitudinal velocity. This adaptive control behavior aligns with the intelligence demonstrated in the earlier analysis of Figure 11(a). This phenomenon also confirms that the collision penalty (Equation 22) effectively guides the agent to make reasonable torque adjustments to avoid excessive penalties, which further demonstrates the intelligence of the IDC-SRAN framework.

26- The torque variation curves in Figure 8(a) show how the four wheels are controlled, but don't analyze the physical implications of these control signals - such as energy efficiency or potential motor wear.

R：Thank you for your comments. We sincerely apologize for the misunderstandings arising from ambiguities in our initial manuscript description. In fact, the FMWR model established in this study excludes motor dynamics. That is, the four wheel torques are not transduced into physical control signals such as motor currents or voltages. Therefore, an declaration regarding this part has been incorporated in the revised manuscript, which have been pasted below for your review. Furthermore, comprehensive consideration of motor dynamics will be addressed in future research.

Clearly, this driving method is more in line with the actual characteristics of the motor and has stronger strategic feasibility compared to Figure 10. To maintain modeling simplicity, the motor model was excluded from the robot dynamics framework developed in this paper. Consequently, this part does not conduct further analysis on motor wear or energy efficiency concerns.

27- The driving force analysis in Figure 8(b) is insightful, but doesn't examine how these forces translate to acceleration profiles and their effects on passenger comfort if this system were deployed in practical applications.

R：Thank you for your comments. The revised manuscript specifically incorporates acceleration curves alongside bar charts illustrating peak and mean acceleration values. Additionally, a brief analysis of the impact of velocity on pedestrian comfort has been conducted, and the revised parts have been pasted below for your review.

Fig 12. The accelerations of ADM-RL and IDC-SRAN in Scene 1.

To quantitatively analyze the impact of IDC-SRAN and ADM-RL on velocity smoothness, acceleration-related data from different algorithms in Scenario 1 are presented in Figures 12 and 13. The acceleration profile of ADM-RL illustrated in Figures 12(a), after entering the crowded area, the robot exhibited drastic velocity fluctuations while maneuvering to avoid pedestrians and proceed toward the goal. Notably, most velocity adjustments were executed within a single discrete time step, resulting in frequent sudden acceleration changes. While these actions demonstrate the intelligence of decision-control, effectively preventing collisions and maintaining correct goal-oriented movement, their practical implementation faces limitations due to actuator dynamic response constraints, thereby compromising the feasibility of realizing such actions in real-world scenarios. Figures 13 the bar chart comparing the mean and maximum absolute acceleration of both IDC-SRAN and ADM-RL. Under identical scenarios, IDC-SRAN demonstrates notably rational acceleration characteristics, with its maximum instantaneous acceleration capped at 1, which is only 8.3% of the peak value by ADM-RL. This substantial reduction in acceleration extremes not only validates the feasibility of IDC-SRAN's decision-control strategy but also enhances pedestrian interaction experience, as smooth acceleration transitions help prevent startling pedestrians. This outcome further corroborates the intelligence of IDC-SRAN in adaptive control and autonomous navigation of robot.

Fig 13. The Bar Chart Comparing The Mean and Maximum Absolute Acceleration of Both IDC-SRAN and ADM-RL

Reviewer #3: The manuscript is well written overall, however, some issues need to be resolved. The comments are as follows:

1 The abstract is concise while informative presenting the background, methods, and a summary of research process, however, there is a lack of key concrete values obtained from the experiment.

R: Thanks for your comments. We fully agree with your professional suggestions on the abstract section. We recognize the key role that the specific presentation of experimental results plays in highlighting the value of the research. The abstract has been comprehensively revised to substantiate the effectiveness of the proposed method with experimental data, incorporating grammatical refinements and enhanced technical clarity. The amended sections of the manuscript are appended below for your reference.

“Reinforcement learning (RL) has demonstrated significant potential in social robot autonomous navigation, yet existing research lacks in-depth discussion on the feasibility of navigation strategies. Therefore, this paper proposes an Integrated Decision-Control Framework for Social Robot Autonomous Navigation (IDC-SRAN), which accounts for the nonlinearity of social robot model and ensures the feasibility of decision-control strategy. Initially, inverse reinforcement learning (IRL) is employed to tackle the challenge of designing pedestrian walking reward. Subsequently, the Four-Mecanum-Wheel Robot dynamic model is constructed to develop IDC-SRAN, resolving the issue of dynamics mismatch of RL system. The actions of IDC-SRAN are defined as additional torque, with actual torque and lateral/longitudinal velocities integrated into the state space. The feasibility of the decision-control strategy is ensured by constraining the range of actions. Furthermore, a critical challenge arises from the state delay caused by model transient characteristics, which complicates the articulation of nonlinear relationships between states and actions through IRL-based rewards. To mitigate this, a driving-force-guided reward is proposed. This reward guides the robot to explore more appropriate decision-control strategies by expected direction of driving force, thereby reducing non-optimal behaviors during transient phases. Experimental results demonstrate that IDC-SRAN achieves peak accelerations approximately 8.3% of baseline methods, significantly enhancing the feasibility of decision-control strategies. Simultaneously, the framework enables goal-oriented autonomous navigation through active torque modulation, attaining a task completion rate exceeding 90%. These outcomes further validate the intelligence and robustness of the proposed IDC-SRAN.”

We reiterate our gratitude for your expert comments.

2 Expand the literature review to provide a more comprehensive background on Autonomous Navigation for Social Robots in similar studies, including recent advancements.

R: We have conducted in-depth discussions, systematically restructured the introduction section, and expanded the literature review in the navigation and sim2real sections to include some studies from the past three years. The specific improvements are as follows.

1. We have incorporated literature on the application of traditional navigation paradigms in open social scenarios and conducted an analysis of their limitations.

“Autonomous navigation, as a core technology for service robots, logistics robots, and other intelligent agents to integrate into human society, is undergoing a paradigm shift from structured environments to dynamic social scenarios, facing the challenge of safe navigation in densely populated environments [2]. Traditional navigation methods based on predefined rules and deterministic optimization, while offering distinct advantages in the interpretability of path generation, often struggle to effectively handle the random behaviors of pedestrian movements [3,4]. The decision freeze represents a fundamental limitation of such approaches in dynamic environments [5,6]. To break through this bottleneck, scholars have proposed an innovative direction of integrating reinforcement learning with traditional planning [7,8]. For example, Pérez et al.[9] by using global path planning to provide waypoint guidance and combining reinforcement learning to handle local pedestrian interactions, the integration of dynamic speed adjustment mechanisms and composite reward functions has successfully addressed the issue of low navigation efficiency in constrained scenarios.”

[2] Ahumada-Newhart V, Eccles J S. Robots for learning: An exploration of teacher roles, perceptions, and challenges in robot-mediated learning. Frontiers in Robotics and AI, 2025, 12.
[3] Wang B, Liu Z, Li Q, & Prorok, A. Mobile robot path planning in dynamic environments through globally guided reinforcement learning. IEEE Robotics and Automation Letters, 2020, 5(4), 6932-6939.
[4] Shi H, Yu W, Madani K. A Review on Social Awareness Navigation for Service Robots. InInternational Conference on Social Robotics, 2024, 143-152.
[5] Zhang P, Chen J J, Jin D, & Jung, S. Service robots in crowded environments: How crowd dynamics shape robotic adoption intention at events, Journal of Hospitality and Tourism Management, 2024, 61, 251-260.
[6] Turkle S. A nascent robotics culture: New complicities for companionship. In Machine ethics and robot ethics Routledge, 2020, 107-116.
[7] Francis A, Pérez-d’Arpino C, et al. Principles and guidelines for evaluating social robot navigation algorithms. ACM Transactions on Human-Robot Interaction, 2025, 14(2), 1-65.
[8] Singamaneni P T, Bachiller-Burgos P, et al. A survey on socially aware robot navigation: Taxonomy and future challenges. The International Journal of Robotics Research, 2024, 43(10), 1533-1572.
[9] Pérez-D’Arpino C, Liu C, et al. Robot navigation in constrained pedestrian environments using reinforcement learning. International Conference on Robotics and Automation (ICRA), 2021, 1140-1146.

2.We have incorporated recent advancements in dynamics-aware RL for robotics and sim-to-real transfer, including several pivotal studies published in 2023-2025.

“In addition, recent studies have enhanced the feasibility of sim2real transfer through perceptual methods [34,35]. For instance, the bird's-eye view semantic conversion method proposed by Reiher [36], and the RetinaGAN image adaptation technique proposed by Ho [37] have demonstrated significant effectiveness.”

[34] Li J, Yang Z, Wang X, et al. Jotr: 3d joint contrastive learning with transformers for occluded human mesh recovery. In Proceedings of the IEEE/CVF International Conference on Computer Vision, 2023, 9110-9121.
[35] Shin J, Jeong H, Rameau F, Kum D. Instagram: Instance-level graph modeling for vectorized hd map learning. IEEE Transactions on Intelligent Transportation Systems, 2025.
[36] Reiher L, Lampe B, Eckstein L. A sim2real deep learning approach for the transformation of images from multiple vehicle-mounted cameras to a semantically segmented image in bird’s eye view. International Conference on Intelligent Transportation Systems (ITSC), 2020, 1-7.
[37] Ho D, Rao K, Xu Z, et al. Retinagan: An object-aware approach to sim2real transfer. International Conference on Robotics and Automation (ICRA), 2021, 10920-10926.

3 Consider clearly stating the research objectives in the introduction to ensure the study's purpose is explicitly defined.

R: Thanks for your comments. A more explicit elucidation of the research objective will facilitate readers' comprehension of the innovative aspects of the research, thereby significantly enhancing the quality of the academic paper. We have revised the introduction section by employing an itemized enumeration to further clarify the research objectives and contributions of this paper. The revised parts of the paper are pasted below for your reference.

“To ensure the feasibility of the decision-control strategy for RL-based socially robots, an Integrated Decision-Control for Social Robot Autonomous Navigation (IDC-SRAN) framework that considers nonlinear dynamics models has been developed in this paper. The contributions of this paper are as follows:

- Pedestrian norm learning based on IRL. To address the issue that pedestrian walking norms are difficult to define through empirical reward functions, IRL is employed to systematically learn pedestrian walking norms. A goal-oriented expert demonstration framework is established, which integrates IRL with a Webots-based training environment for interactive exploration. This methodology enables the identification of both explicit and implicit rewards associated with pedestrian walking norms, thereby enhancing robotic social awareness in complex societal environments.
- Establishment of an Integrated Decision-Control Framework. By introducing a nonlinear dynamic model for the Four-Mecanum-Wheel Robot (FMWR), this study effectively resolves the dynamic mismatch issue prevalent in existing research. Furthermore, the system's actions are defined as incremental torques for the four wheels, which can be directly applied to actual robotic control units. This approach achieves an integrated decision-control for robot autonomous navigation, while enhancing the feasibility of decision-control strategies.
- Design of Driving-Force-Guided Reward Function. Considering the challenge posed by state delays resulting from transient characteristics of the model in reward function design, this study innovatively integrates features of driving force into the reward mechanism. By rewarding driving forces aligned with correct directions, the framework guides robots to conduct proper exploration during transient model phases. This approach ensures task efficiency and safety while significantly reducing the occurrence of suboptimal behaviors.”

4 Provide figures of the constructed scenario and the experiment process.

R：Thank you for your comments. The initial manuscript version indeed omitted the technical details on the simulator parameters. In the revised version, we have incorporated a new section that provides comprehensive elaboration of environmental details, facilitating readers' comprehension of pedestrian behavioral patterns within the simulated environment as well as its specific environmental parameters. The revised parts of this paper have been pasted below for your review.

To train the proposed IDC-SRAN framework and validate its autonomous navigation capabilities, this study leveraged the Webots simulation platform to construct a testing and validation environment that replicates the real-world environment of the ETH dataset, as illustrated in Figure 6.

Fig 6. Simulation Environment for Testing and Validation (Scene 1). (a) Real-World Environment of the ETH Dataset. (b) Simulation Environment with Webots

Figure 6(a) depicts the real-world environment of the ETH dataset, and Figure 6 (b) presents the testing and validation simulation environment with the Webots simulation platform. The white lines represent walls enclosing a rectangular structure in the lower-left area, resembling commercial facilities in real-world environments with pedestrian-impermeable boundaries. It is noteworthy that the simulation environment does not constitute a 1:1 replication of the real-world environment. Specifically, parked vehicles along road edges are treated as pedestrian-impermeable boundaries in the simulation, and the depth of the lower-right corner is deliberately deepened. This design modification reduces corner width while increasing its length, thereby enhancing the operational complexity for robots to pass through the corner. Such modification further facilitate the training and validation of rationality in autonomous navigation decision-control strategies. The simulated environment measures 21.6 meters in width and 17.4 meters in length, with the commercial facilities in the lower-left area measures 15.5 meters in width and 7.7 meters in length, containing a total of 15 pedestrians.

Fig 7. Additional verification environment for Real-World Corridor (Scene 2)

Furthermore, this study is grounded in the assumption of global observability, neglecting the research on the perception module. Consequently, IDC-SRAN remains constrained to simulation environments and faces significant challenges in practical deployment on real-world robotic platforms. Notably, limited scene generalization capability emerges as one of the critical obstacles in practical implementation of autonomous navigation decision-control systems. Therefore, an additional verification environment was developed based on existing conditions, which is shown in Figure 7, precisely aligned with a real-world corridor to validate the generalization performance of IDC-SRAN. The additional verification environment features a rectangular configuration with a length of 12 meters and a width of 8.5 meters, containing a total of 10 pedestrians. The movement of all pedestrians within the environment is governed by the social force model, which continuously calculates their speed and position at each discrete time step. Motion control in the simulated environment is ultimately executed through the Supervisor node of the Webots platform, enabling precise simulation of pedestrian dynamics.

5 Elaborate on why IDC-SRAN could ensure the intelligence and feasibility of social robot autonomous navigation. Including potential factors influencing the experimental outcomes would strengthen the discussion.

R：Thank you for your comments. We sincerely apologize for the overly ambiguous analysis and insufficient quantitative metrics in the experimental section of the initial manuscript, which compromised the clarity of demonstrating IDC-SRAN's enhanced intelligence and feasibility for social robot autonomous navigation, thereby affecting the overall quality of the study. In the revised manuscript, we have added bar charts illustrating the mean and maximum acceleration values (Figure 13), along with comparing collision rates and completion rates across different scene (Table 1). These statistical additions will provide readers with intuitive insights into the core contributions of this study. Furthermore, we have conducted more comprehensive and detailed analyses of simulation curves to ensure clearer comprehension of the research conclusions. Once again, we sincerely appreciate your valuable feedback, which has been instrumental in enhancing the manuscript's quality. Due to the wide coverage of the revised content, please refer to Chapter 4 of the manuscript for specific details.

6 It is recommended to perform validation experiments on a real test platform to evaluate the autonomous navigation capabilities of the proposed Framework.

R：Thank you for your comments. In the initial version, we neglected to address the limitations of simulated environments. It is undeniable that conducting experiments on physical test platforms would further enhance the paper's quality and strengthen the credibility of conclusions. However, practical deployment of IDC-SRAN currently faces significant challenges due to hardware constraints, including the absence of perception module and the motor control algorithm module (torque conversion into motor control electrical signals). Therefore, the revised manuscript analyzes common Sim2Real challenges and incorporates additional simulation experiments employing a compromise-based verification methodology. We fully acknowledge the significant discrepancies between this approach and actual physical validation. In future research, we commit to advancing IDC-SRAN toward practical deployment by resolving critical hardware limitations through the development of integrated perception systems and advanced motor control solutions. Thank you again for your meticulous review, as it has been instrumental in refining our paper. The revised content is pasted below for your review.

“This study focuses on enhancing the feasibility of robotic decision-control strategies through the deployment of dynamics models, while not addressing perception-related research. Consequently, practical deployment of the current IDC-SRAN framework still faces challenges. Considering that Sim2Real challenges for decision-control frameworks primarily stem from aspects such as real-time, feasibility and scene generalization, and the feasibility has been proven. Therefore, this paper conducts multiple repeated simulation experiments in Scene 1 and 2, complemented by statistical analysis of computation time (including the combined computational time of environmental states and policy networks), to indirectly validate the framework’s practical applicability. It's no doubt that this compromise validation approach has limitations: it does not account for unknown disturbances in sensor perception and overlooks discrepancies between simulated conditions (robot models, pedestrian kinematics, environments) and real-world implementations. These gaps will be addressed in future research.

Table 1. Collision Rates and Completion Rates across Different Scene

| Scene | Method | Computing time | Completion rate | Collision rate | |
| --- | --- | --- | --- | --- | --- |
|  |  |  |  | Person | Wall |
| Scene 1(15) | ADM-RL | 0.009 s | 94 | 4 | 2 |
|  | IDC-SRAN | 0.011 s | 91 | 7 | 2 |
| Scene 2(10) | ADM-RL | 0.009 s | 92 | 5 | 3 |
|  | IDC-SRAN | 0.011 s | 90 | 6 | 4 |

Table 1 presents the pass rates and average computation times of different algorithms under repeated simulations across multiple scene. Specifically, Scene 1 involves 15 pedestrians with randomized initial and goal positions, while Scene 2 includes 10 such pedestrians. In all repeated simulation, pedestrian motion trajectories remain distinct. This methodology ensures the randomicity and statistical validity of simulation experiments through systematic randomization. It can be observed that compared to ADM-RL, the completion rate of IDC-SRAN incorporating the dynamic model exhibits a slight decrease. This stems from the fact that the motions of IDC-SRAN adhere to dynamic characteristics, eliminating abrupt action adjustments. Consequently, delayed collision avoidance maneuvers may occur, leading to an increased collision rate. However, when weighed against the substantial reduction in acceleration magnitudes (indicative of enhanced feasibility), this marginal loss in completion rate remains operationally acceptable. Simultaneously, the expanded state space (incorporating velocity and actual torques) results in moderately elevated computational demands. Nevertheless, the computational speed remains within practical limits, enabling real-time given the simulation step size of 0.4 seconds. Notably, both ADM-RL and IDC-SRAN demonstrate scenario generalization capabilities, as the completion rate of Scene 2 did not show a significant decrease.”

Reviewer #4: Suggestion for modification:

Further explore how to improve the effectiveness of IRL.

R：Thank you for your comments. In the revised manuscript, we further explore how to improve the effectiveness of IRL. The revised content is pasted below for your review.

“The future work of this study will focus on refining the model: simplified model assumptions often lead to accuracy degradation, which adversely impacts control performance. Given that enhancing data quality and quantity is critical for advancing the effectiveness of Inverse Reinforcement Learning (IRL), this will remain a key priority in our future research. Additionally, this study will further investigate the previously overlooked visual module to enable practical deployment of the decision-control algorithms.”

Design a more comprehensive reward function to better meet the needs of autonomous navigation for social robots.

R：Thank you for your comments.

“Furthermore, discrete rewards such as collision penalties $R_{coll}$ and task completion rewards $R_{com}$ are jointly deployed within the IDC-SRAN framework, which can be expressed as:

$R_{coll}=\left\{\begin{matrix} 1, & \Delta d<0.5m \\ 0.5, & \Delta d<1.2m \\ 0, & else \end{matrix}\right.$ (22)

$R_{com}=\left\{\begin{matrix} 1, & \parallel\vec{d}\parallel<0.1m \\ 0, & else \end{matrix}\right.$ (23)

In summary, the reward function R of IDC-SRAN can expressed as,

$R=R_{f}+c_{3}R_{coll}+c_{4}R_{com}+c_{5}R_{s}$ (24)

$R_{f}=c_{1}R_{G}+c_{2}R_{o}$ (25)

Where c_i_ are referred to as the reward weighting constants.”

Conduct experimental verification on actual robot platforms to validate the effectiveness of the IDC-SRAN framework in practical applications.

R：Thank you for your comments. In the initial version, we neglected to address the limitations of simulated environments. It is undeniable that conducting experiments on physical test platforms would further enhance the paper's quality and strengthen the credibility of conclusions. However, practical deployment of IDC-SRAN currently faces significant challenges due to hardware constraints, including the absence of perception module and the motor control algorithm module (torque conversion into motor control electrical signals). Therefore, the revised manuscript analyzes common Sim2Real challenges and incorporates additional simulation experiments employing a compromise-based verification methodology. We fully acknowledge the significant discrepancies between this approach and actual physical validation. In future research, we commit to advancing IDC-SRAN toward practical deployment by resolving critical hardware limitations through the development of integrated perception systems and advanced motor control solutions. Thank you again for your meticulous review, as it has been instrumental in refining our paper. The revised content is pasted below for your review.

“This study focuses on enhancing the feasibility of robotic decision-control strategies through the deployment of dynamics models, while not addressing perception-related research. Consequently, practical deployment of the current IDC-SRAN framework still faces challenges. Considering that Sim2Real challenges for decision-control frameworks primarily stem from aspects such as real-time, feasibility and scene generalization, and the feasibility has been proven. Therefore, this paper conducts multiple repeated simulation experiments in Scene 1 and 2, complemented by statistical analysis of computation time (including the combined computational time of environmental states and policy networks), to indirectly validate the framework’s practical applicability. It's no doubt that this compromise validation approach has limitations: it does not account for unknown disturbances in sensor perception and overlooks discrepancies between simulated conditions (robot models, pedestrian kinematics, environments) and real-world implementations. These gaps will be addressed in future research.

Table 1. Collision Rates and Completion Rates across Different Scene

| Scene | Method | Computing time | Completion rate | Collision rate | |
| --- | --- | --- | --- | --- | --- |
|  |  |  |  | Person | Wall |
| Scene 1(15) | ADM-RL | 0.009 s | 94 | 4 | 2 |
|  | IDC-SRAN | 0.011 s | 91 | 7 | 2 |
| Scene 2(10) | ADM-RL | 0.009 s | 92 | 5 | 3 |
|  | IDC-SRAN | 0.011 s | 90 | 6 | 4 |

Table 1 presents the pass rates and average computation times of different algorithms under repeated simulations across multiple scene. Specifically, Scene 1 involves 15 pedestrians with randomized initial and goal positions, while Scene 2 includes 10 such pedestrians. In all repeated simulation, pedestrian motion trajectories remain distinct. This methodology ensures the randomicity and statistical validity of simulation experiments through systematic randomization. It can be observed that compared to ADM-RL, the completion rate of IDC-SRAN incorporating the dynamic model exhibits a slight decrease. This stems from the fact that the motions of IDC-SRAN adhere to dynamic characteristics, eliminating abrupt action adjustments. Consequently, delayed collision avoidance maneuvers may occur, leading to an increased collision rate. However, when weighed against the substantial reduction in acceleration magnitudes (indicative of enhanced feasibility), this marginal loss in completion rate remains operationally acceptable. Simultaneously, the expanded state space (incorporating velocity and actual torques) results in moderately elevated computational demands. Nevertheless, the computational speed remains within practical limits, enabling real-time given the simulation step size of 0.4 seconds. Notably, both ADM-RL and IDC-SRAN demonstrate scenario generalization capabilities, as the completion rate of Scene 2 did not show a significant decrease.”

Improve the references in the paper and ensure that the citation format is correct.

R: We sincerely appreciate your professional suggestions regarding the reference section. We have systematically undertaken comprehensive revisions to ensure consistent application of Vancouver Style for reference management throughout the manuscript. Thank you again for your meticulous review, which has been invaluable in improving our paper.
